# The Transcriptional Regulator CzcR Modulates Antibiotic Resistance and Quorum Sensing in *Pseudomonas aeruginosa*

**DOI:** 10.1371/journal.pone.0038148

**Published:** 2012-05-29

**Authors:** Guennaëlle Dieppois, Véréna Ducret, Olivier Caille, Karl Perron

**Affiliations:** Microbiology Unit, Department of Botany and Plant Biology, Sciences III, University of Geneva, Switzerland.; Vrije Universiteit Brussel, Belgium

## Abstract

The opportunistic pathogen *Pseudomonas aeruginosa* responds to zinc, cadmium and cobalt by way of the CzcRS two-component system. In presence of these metals the regulatory protein CzcR induces the expression of the CzcCBA efflux pump, expelling and thereby inducing resistance to Zn, Cd and Co. Importantly, CzcR co-regulates carbapenem antibiotic resistance by repressing the expression of the OprD porin, the route of entry for these antibiotics. This unexpected co-regulation led us to address the role of CzcR in other cellular processes unrelated to the metal response. We found that CzcR affected the expression of numerous genes directly involved in the virulence of *P. aeruginosa* even in the absence of the inducible metals. Notably the full expression of quorum sensing 3-oxo-C12-HSL and C4-HSL autoinducer molecules is impaired in the absence of CzcR. In agreement with this, the virulence of the *czcRS* deletion mutant is affected in a *C. elegans* animal killing assay. Additionally, chromosome immunoprecipitation experiments allowed us to localize CzcR on the promoter of several regulated genes, suggesting a direct control of target genes such as *oprD*, *phzA1* and *lasI*. All together our data identify CzcR as a novel regulator involved in the control of several key genes for *P. aeruginosa* virulence processes.

## Introduction

Two-component signal transduction systems (TCS) are the most important mechanisms used by bacteria to detect and respond to changing environmental conditions and stresses. Upon sensing external or internal stimuli, the TCS phosphorylation cascade enables the bacterial cells to modulate gene expression and to adapt their physiology in a specific and rapid manner [1]. The two partners of a classical TCS are the sensor histidine kinase (HK) and the response regulator (RR). The HK sensor is usually a membrane-spanning protein which upon signal recognition, dimerizes and autophosphorylates on a conserved histidine residue using ATP. The phosphoryl group is then transferred to an aspartate residue on the cognate receiver domain of the RR protein. Following phosphorylation, the output domain becomes active to mediate the adapted response. The majority of RR proteins possess an output domain containing DNA-binding activities, allowing them to directly modulate gene transcription [2]. In the past few years, the emerging picture of cross-talk activity and coordination between different TCS raised on the horizon a more complex view of two-component signal transduction [3], [4], [5].

Approximately 130 genes encoding for TCS modules have been identified in the genome of *P. aeruginosa* [6], [7]. This huge number indicates that *P. aeruginosa* possesses complex regulatory strategies to face environmental challenge and could explain the ubiquity of this organism. Indeed, *P. aeruginosa* is one of the most versatile bacteria, capable of living in many diverse environments [8], [9]. By virtue of its vast adaptability, this Gram-negative bacterium is also a major opportunistic pathogen, causing serious nosocomial infections, severe problems in cystic fibrosis and immunocompomised patients as well as in burn victims [10]. Furthermore, *P. aeruginosa* is intrinsically resistant to multiple classes of antimicrobial compounds. This is a major cause of therapeutic failure in the treatment of infections [11].

We previously characterized the metal-inducible TCS CzcRS in this bacterium [12], [13]. In the presence of Zn, Cd, Co, or indirectly in the presence of Cu, the metal-inducible TCS CzcRS is activated. CzcR then promotes the expression of the metal efflux pump CzcCBA. Additionally, CzcR down-regulates the expression of the OprD porin, the route of entry of carbapenem antibiotics [14], [15]. As the result of this co-regulation, the presence of Zn, Cd, Co, or Cu in the environment render *P. aeruginosa* resistant to both trace metals and carbapenems [12], [13]. Carbapenem represents an important class of antibiotics active against both Gram-negative and Gram-positive bacteria. They are often used as the last choice of treatment against *P. aeruginosa* and resistance to these antibiotics is a major worldwide problem [16]. In *P. aeruginosa*, the main and most frequent mechanism of carbapenem resistance is a decrease in OprD expression, occurring at the transcriptional or post-transcriptional level [17], [18]. This unusual co-regulation mechanism between metal and carbapenem resistance is of particular concern since clinical strains of *P. aeruginosa* are not genetically different from their environmental counterparts [19], [20]. Environmental metal pollutants might therefore have a direct effect on the physiology of this pathogen.

In addition to its strong ability to resist many different antimicrobial compounds, *P. aeruginosa* possesses intricate regulatory quorum sensing systems (QS) that control, in a cell density-dependent manner, the expression of more than a hundred genes, including those required for virulence factor expression and biofilm formation, [21], [22]. The two major QS are the interconnected *las* and the *rhl* systems, the *las* system controlling the expression of the *rhl* system (reviewed in [23]). A third cell-to-cell signaling pathway has been characterized in *P. aeruginosa* [24]. It involves quinolone molecules (Pseudomonas Quinolone Signal, also called PQS) and could account for an additional level of control of virulence factor expression [25], [26].

Furthermore The QS circuit is strongly integrated within other cellular processes and environmental or internal stimuli are required for full expression of target genes [27]. For instance, several factors are known to positively and negatively modulate QS under different conditions [23], [28]. In the present paper, we found that CzcR, the regulatory protein for the CzcRS TCS, is required for full expression of QS-regulated genes. Moreover the results showed that CzcR is able to bind directly to several promoters linking metal stress, antibiotic resistance and quorum sensing in *P. aeruginosa.*

## Results

### CzcR negatively regulates pyocyanin biosynthesis

**Figure 1:**
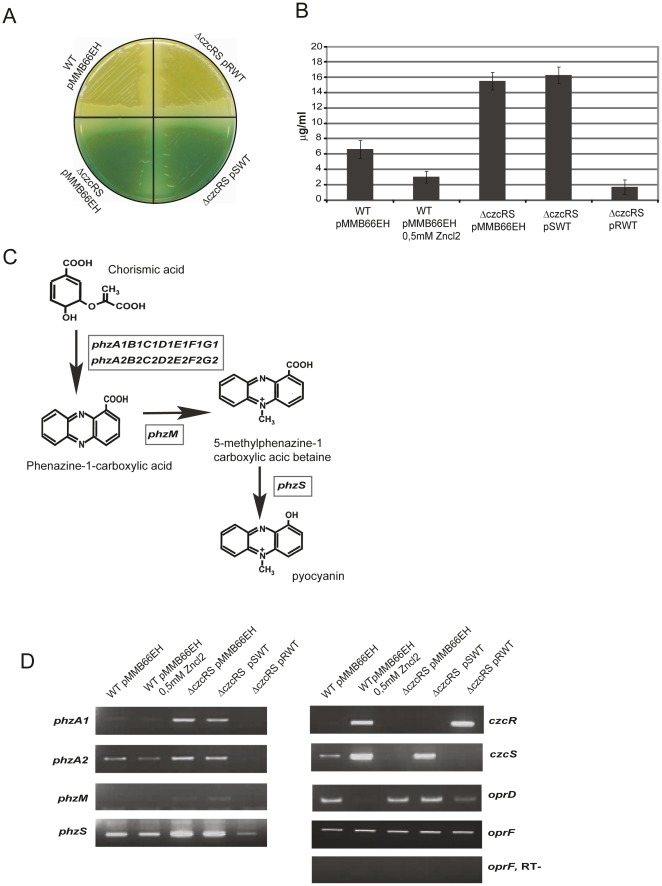
CzcR represses pyocyanin biosynthesis. A) Pyocyanin production on KingA agar plate in the wt and the Δ*czcRS* complemented strains. pMMB66EH: empty vector; pSWT: *czcS* gene; pRWT: *czcR* gene. B) Quantification of pyocyanin produced in KingA medium by the wt and the Δ*czcRS* complemented strains. The experiment was performed three times and standard deviations (errors bars) are indicated. C) Schematic representation for the synthesis of pyocyanin, according to [29]. Genes involved in the different steps are boxed. D) Transcription of the wt and the Δ*czcRS* complemented strains analyzed by semi-quantitative RT-PCR. Negative controls lacked reverse transcriptase (RT-).

In our previous work, we constructed a *P. aeruginosa* PAO1 strain deleted for the metal-inducible two-component system *czcRS* [12]. Unexpectedly, while both strains displayed a similar growth rate (data not shown), the Δ*czcRS* double knockout mutant exhibited a pronounced blue-green pigmentation, diffusing into LB or King A agar plate medium, compared to the wild type strain (Fig. 1A). This color is a characteristic of the phenazine-derived pigment pyocyanin. Assay of this pigment confirmed that pyocyanin levels were more than twofold higher in Δ*czcRS* mutant than in the wild type (Fig. 1B). Moreover, in the presence of zinc, a condition where the CzcRS system is activated and strongly expressed [13], pyocyanin production was significantly decreased in wild type cells. Altogether these observations suggest that the CzcRS system may play a role in down-regulating pyocyanin biosynthesis. To verify this hypothesis and define which component between the sensor and the regulator is involved in this regulation, we reintroduced into the Δ*czcRS* strain either the *czcS* (pSWT) or the *czcR* (pRWT) gene cloned under the control of the IPTG-inducible *tac* promoter in plasmid pMMB66EH (see materials and methods). Like Δ*czcRS,* Δ*czcRS-*pSWT exhibited a marked blue-green coloration on plates and increased levels of pyocyanin compared to wild type (Fig. 1A and B). Interestingly, the expression of CzcR in Δ*czcRS*-pRWT drastically decreased pyocyanin levels and completely rescued the wild type yellow coloration (Fig. 1A and B), suggesting that CzcR is a negative regulator of pyocyanin synthesis.

In *P. aeruginosa*, pyocyanin biosynthesis involves two homologous core operons, *phzA1B1C1D1E1F1G1* and *phzA2B2C2D2E2F2G2,* in phenazine-1-carboxylic acid (PCA) [29]. PCA is then processed into pyocyanin by the products of two additional genes, *phzM* and *phzS* (Fig. 1C). Since CzcR negatively regulates pyocyanin synthesis, we wondered whether it affects the transcription of these biosynthetic genes. RT-PCR experiments were therefore performed to monitor the amount of *phzA1*, *phzA2*, *phzM* and *phzS* mRNAs (Fig. 1D). In agreement with the previously observed phenotype (Fig. 1A), the Δ*czcRS* mutant (Δ*czcRS-*pMMB66EH) strongly overexpresssed the *phzA1* operon compared to the wt strain (Fig. 1D). These phenotypes were complemented by the *czcR* gene added in *trans* on a plasmid (pRWT) but not by the *czcS* gene (pSWT). The second pyocyanin operon *phzA2,* as well as the *phzS* and the *phzM* genes are also slightly regulated since the overexpression of CzcR (Δ*czcRS-*pRWT) decreased the amount of mRNA level below the wt (Fig. 1D). As expected, addition of Zn to the medium repressed the expression of both the *phzA1* and *phzA2* operons and the *phzS* gene. In this case however, we observed a weaker repression compared to the effect mediated by the CzcR protein overexpressed using IPTG. This suggested a more complex regulatory process occurring in the presence of Zn.

As a control, we analyzed in parallel *czcR*, *czcS* and the porin gene *oprD,* a known target repressed by CzcR [12], [13]. As expected, in the wt strain, *czcR* and *czcS* mRNA levels increased in the presence of zinc. Furthermore, consistent with our previous works [12], [13], *oprD* mRNA levels were drastically affected by zinc as well as by CzcR expression. Altogether these results suggest that CzcR is a transcriptional repressor of *phzA1*, *phzA2* operons and *phzS* involved in the pyocyanin biosynthesis and *oprD* involved in the import of basic amino acids and carbapenem antibiotics (Fig. 1D and [13]).

### Deletion of CzcR affects QS-regulated phenotypes

**Figure 2:**
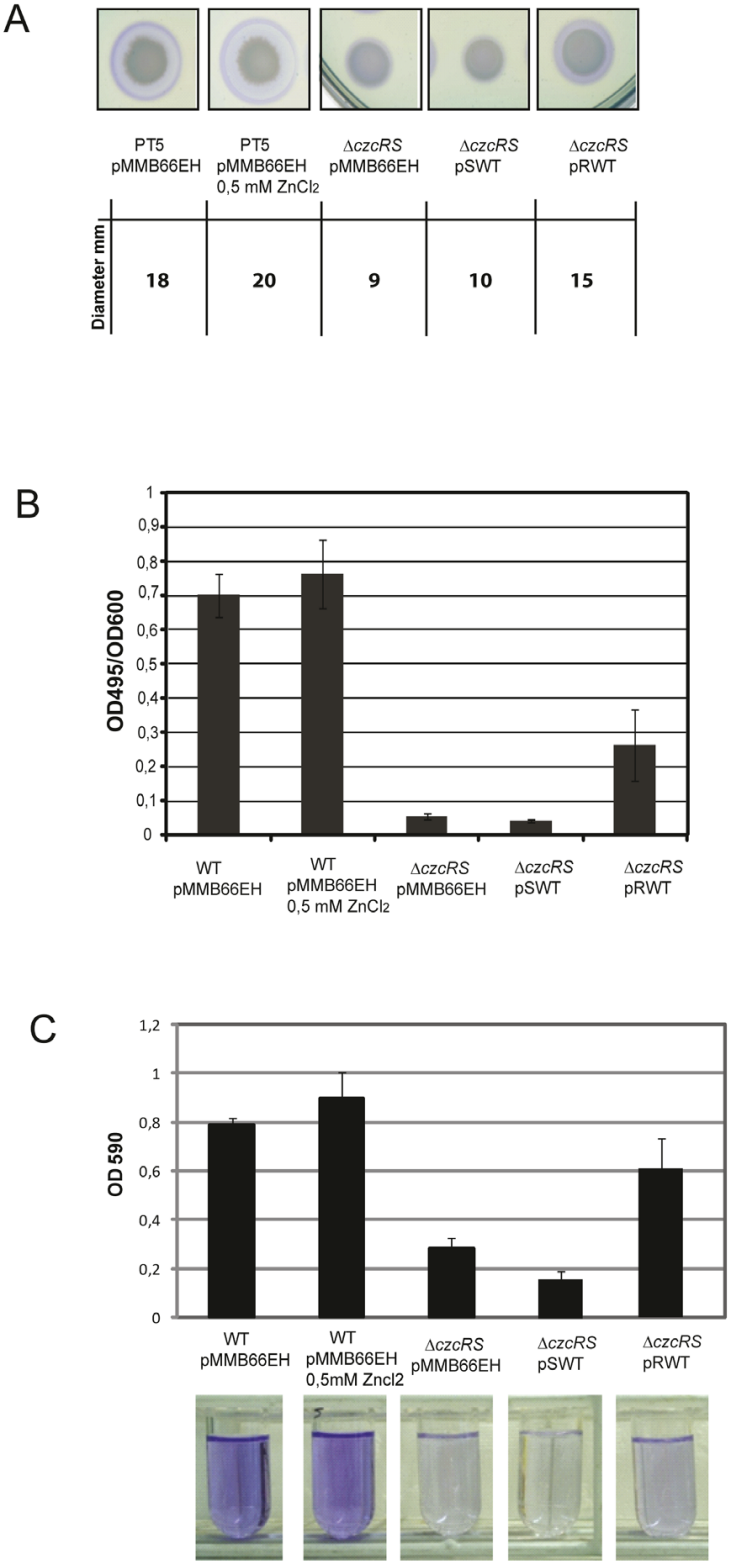
CzcR is required for virulence factor production and biofilm formation. Rhamnolipids, elastase production and biofilm formation in the wt and the Δ*czcRS* complemented strains. pMMB66EH: empty vector; pSWT: *czcS* gene; pRWT: *czcR* gene A) Rhamnolipid production on SW blue plate assay. The blue halo is due to the presence of rhamnolipids produced by the colony. Diameters of these halos are indicated below the figure. B) Elastase production determined by the Elastine-Congo Red assay. The determination was performed three times independently and standard deviations (errors bars) are indicated. C) Biofilm formation in M9 minimal medium on polypropylene tubes after 8 h of static growth. Biofilms were stained with cristal violet and quantified at OD_590_ after solubilization in acetic acid. Quantification was performed in three independent experiments and standard deviations (errors bars) are indicated.

Since pyocyanin synthesis is under quorum sensing (QS) control [30], we investigated whether CzcR influences the production of elastase and rhamnolipids, two other QS-controlled virulence factors. In agreement with a role of CzcR as a positive regulator of QS, rhamnolipid production was dramatically impaired in the Δ*czcRS* mutant compared to a wild type strain, as determined by SW blue plate assay [31] (Fig. 2A). Using the elastin-Congo red (ECR) assay [32] we also found that the LasB elastase activity in supernatants of the Δ*czcRS* mutant was more than 10-fold less than that found in wild-type supernatants (Fig. 2B). Another important QS-dependent phenotype related to *P.aeruginosa* pathogenesis, persistence and colonization is its ability to form biofilm. Similar to the previous observed phenotypes, biofilm formation in polypropylene tubes was strongly impaired in the Δ*czcRS* mutant relative to wild type (Fig. 2C).

The production of rhamnolipids, elastase and biofilm formation could be partially but significantly restored in the Δ*czcRS* mutant by complementation with the *czcR* gene on a plasmid (pRWT) but not by the *czcS* gene. This result confirms the requirement of CzcR protein for the full induction of these QS-regulated phenotypes (Fig. 2A and 2B). Collectively these data indicate that CzcR plays an important role in the control of virulence and biofilm formation of *P. aeruginosa*. However, despite the observation of a slight repeatable increase, the production of rhamnolipids, elastase and biofilm formation did not appear to be significantly affected by the presence of Zn in the medium. These data suggest that a basal level of CzcR, in the absence of Zn, is sufficient for full expression of these phenotypes.

### CzcR stimulates the expression of QS signaling pathways

**Figure 3:**
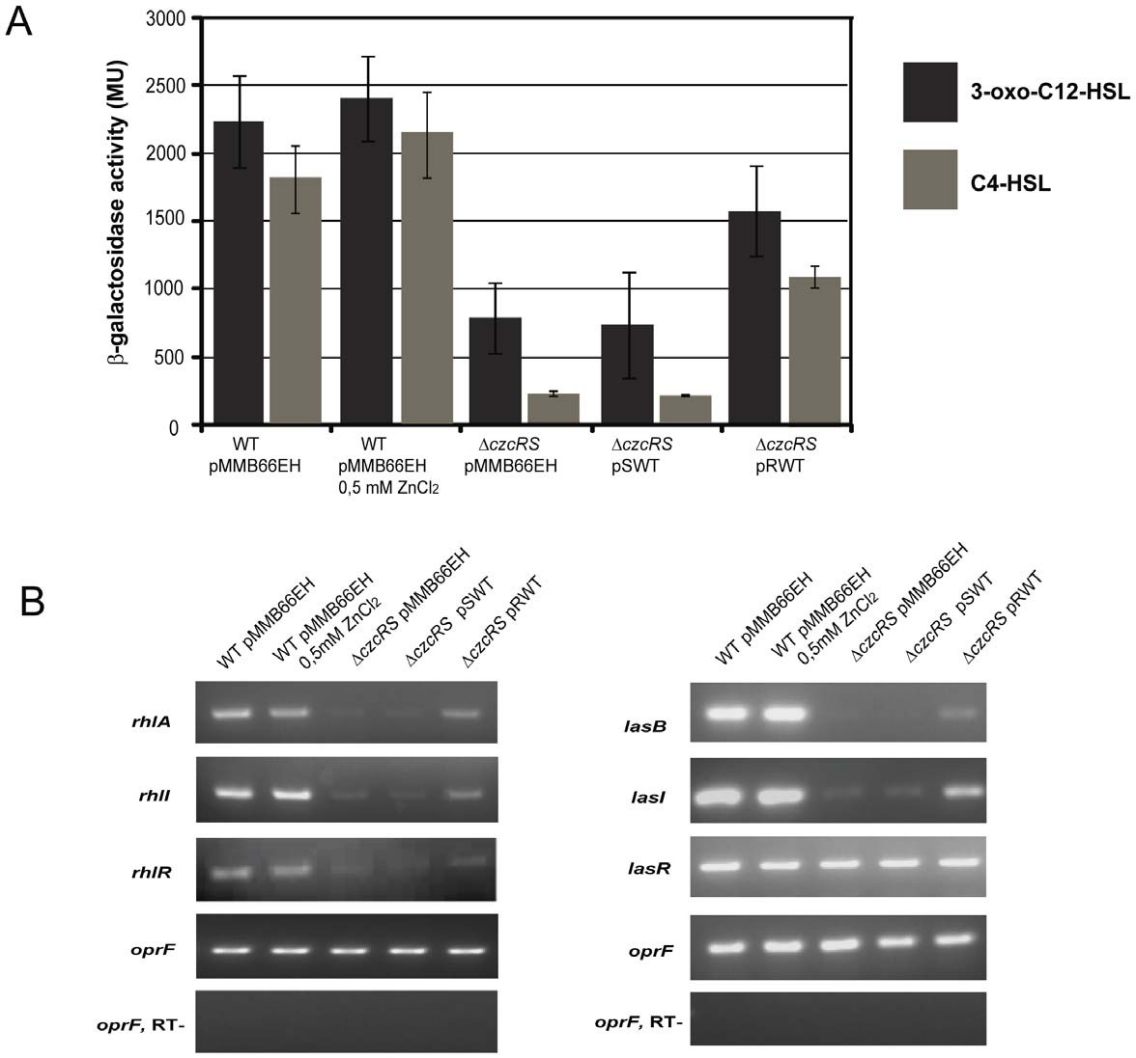
CzcR stimulates the expression of QS genes. Autoinducer production and expression of QS genes in the wt and the Δ*czcRS* complemented strains. pMMB66EH: empty vector; pSWT: *czcS* gene; pRWT: *czcR* gene. A) Quantification of AHL in the supernatant of LB culture using the beta-galactosidase reporter assay. Error bars represent the standard deviations of three independent quantifications. B) Semi-quantitative RT-PCR analysis of the *las* and *rhl* QS transcripts for the wt and the Δ*czcRS* complemented strains. Negative controls lacked reverse transcriptase (RT-).

The fact that the deletion of CzcR affects the expression of several QS-controlled genes suggested that it could act at the stage of QS regulators or autoinducer expression. We first monitored levels of the two major signal molecules, N-3-oxo-dodecanoyl-L-homoserine lactone 3-oxo-C12-HSL (3-oxo-C12-HSL) and N-butanoyl-L-homoserine lactone (C4-HSL) autoinducers, in the filtered supernatant of early stationary phase culture [33], [34] (Fig. 3A). Our results showed a drastic decrease in the amount of both autoinducers in the Δ*czcRS* mutant compared to the wild type. No significant changes in 3-oxo-C12-HSL and C4-HSL autoinducer accumulation, however, were observed in a wild type strain grown in the presence or absence of zinc. Wild type autoinducer levels were partially restored in Δ*czcRS* complemented in *trans* by pRWT, whereas they were not rescued by pSWT (Fig. 3A), showing that both 3-oxo-C12-HSL and C4-HSL production are impaired in the absence of CzcR.

Since autoinducer synthases and regulators are themselves controlled by QS, we monitored by RT-PCR the expression of the *las* and *rhl* QS systems (*lasI, lasR* and *rhlI, rhlR* genes), along with the genes *lasB* (encoding elastase) and *rhlA* (encoding a rhamnolipid biosynthesis enzyme) directly regulated by these systems (Fig. 3B). We observed a significant down-regulation in the expression of *rhlA*, *rhlI*, *rhlR* as well as *lasB* and *lasI* in the Δ*czcRS* mutant compared to the wild type. In coherence with the phenotypes shown in figure 2, the decrease in mRNA levels was partially rescued by overexpression of *czcR* in *trans* but not by the complementation with *czcS*. Surprisingly, *lasR* mRNA levels were not affected in the Δ*czcRS* mutant. In agreement with the absence of effect on autoinducer levels (Fig. 3A), addition of zinc to a wild type strain did not noticeably modulate the transcription levels of the observed genes.

Since the Las and the Rhl quorum sensing systems are interconnected with the PQS system, we also investigated the amount of *pqsH* mRNA. PqsH is the enzyme involved in the last step of PQS synthesis. Consistent with the positive control of *pqsH* by LasR [35], we observed a strong decrease in *pqsH* transcription in the *ΔczcRS* mutant, a deficiency that could be complemented with *czcR* but not by *czcS* (data not shown).

In the absence of external stimuli, low basal levels of functional regulator proteins have already been observed for several TCS systems [36], [37], [38], [39]. Our results suggest that a low level of the CzcR protein might trigger important cellular responses in the absence of its inducible metal by permitting the proper transcription of the *lasI, rhlI* and *rhlR* quorum sensing regulatory genes.

### CzcR is required for full virulence in *Caenorhabditis elegans*

**Figure 4:**
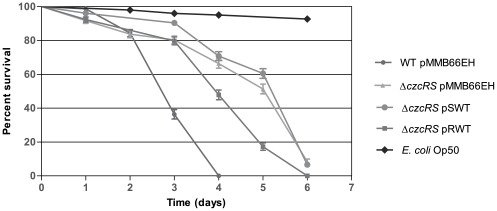
CzcR is required for the full virulence of *P. aeruginosa*. Killing of *C. elegans* fed with wild-type *P. aeruginosa* or with the Δ*czcRS* complemented strains (pMMB66EH: empty vector; pSWT: *czcS* gene; pRWT: *czcR* gene). *E. coli* OP50 was used as the non-pathogenic strain. The percentage of living worms, scored up to 6 days at 24 h-intervals, was plotted using GraphPad Prism 5 software. Data represent the average of three independent experiments. The comparison between the survival curves of WT pMMB66EH and *ΔczcRS* pMBB66EH, *ΔczcRS* pMMB66EH and *ΔczcRS* pRWT was performed by means of two different tests: the Log-rank (Mantel-Cox) test and the Gehan-Breslow-Wilcoxon test. Both tests showed that the curves were significantly different with a P value below 0.0001. The *ΔczcRS* pMMB66EH and *ΔczcRS* pSWT survival curves were also compared. This comparison gave a P value of 0.07 with the Log-rank (Mantel-Cox) test and 0.03 with the Gehan-Breslow-Wilcoxon test, indicating that these two curves are not significantly different.

Since CzcR is involved in the expression of QS-regulated virulence factors, we wondered whether this regulator is important for the pathogenicity of *P. aeruginosa.* To this aim, we used the nematode *Caenorhabditis elegans* as a model of infection [40]. *P. aeruginosa* killing of the worm *C. elegans* is a multifactorial process involving both toxin-mediated mechanisms and a slower infectious process requiring bacterial proliferation within the gut [41]. The *P. aeruginosa* PAO1 strain used in this study is not cytotoxic and only slow killing was detected. In our assay, an infection with the wild type strain killed 50% of the population within circa 3 days of contact with bacteria and all worms were dead after 4 days (Fig. 4). In contrast, the virulence of the Δ*czcRS* mutant was significantly reduced since 50% of the worms were still alive after 5 days of contact. The virulence of the Δ*czcRS* mutant strain complemented with *czcR* on the pRWT plasmid was partially restored since 50% of the worms were dead after 4 days and less than 20% of the worm population was alive after 5 days, while no complementation was observed with the *czcS* gene (Fig. 4). The effect is statistically relevant as determined using the Log-rank and Gehan-Breslow-Wilcoxon tests (P value<0.0001). Deletion of this TCS did not completely abolish virulence. The delay we observed might suggest that the *ΔczcR* only partially affects the global virulence of *P. aeruginosa*. The observed result cannot account for a decrease of the bacterial number since no growth defect in plate and in liquid medium was observed in the Δ*czcRS* complemented strains (data not shown). A control with the non-virulent reference strain *E. coli* OP50 (non-pathogenic for *C. elegans*), showed that worm viability was stable after 5 days of contact with these bacteria (Fig. 4). Altogether these results strongly suggest that CzcR, by acting positively on quorum sensing gene expression, plays an important role in the pathogenicity of *P. aeruginosa.*

### CzcR is associated with *oprD* and *phzA1* promoters

**Figure 5:**
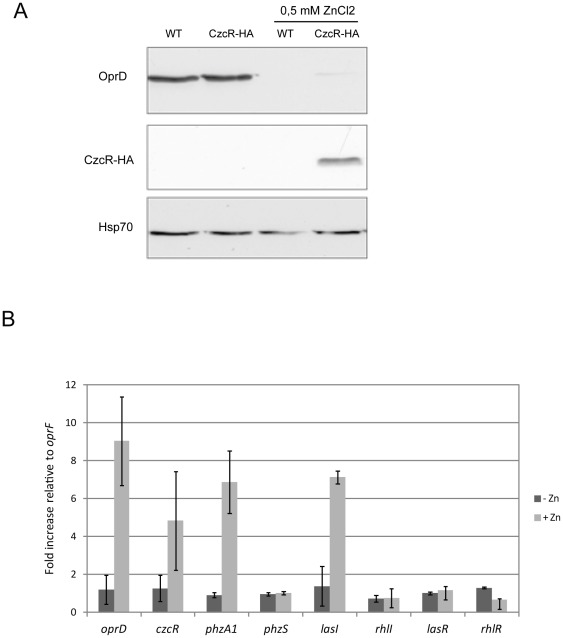
CzcR is associated to the *oprD*, *phzA1* and *czcR* promoters. A) Western blot analysis of the wild-type and the CzcR-HA strain carrying the HA-tagged version integrated into the chromosome. Total proteins from the wild-type or the CzcR-HA strain grown in the presence or absence of 0.5 mM ZnCl_2_ in LB medium were analyzed by western blot. Antibodies used for the analysis are indicated in the left of the panel. B) Chromosome immunoprecipitation performed on the CzcR-HA strain using anti-HA antibody. Experiment was performed on culture grown in the absence or in the presence of 0.5 mM ZnCl_2_, as indicated. Results represent the average of 3 independent experiments and standard deviations are indicated.

We showed that CzcR plays a negative role in the transcriptional regulation of the *oprD* gene coding for the porin involved in the entry of basic amino acids and of carbapenem antibiotics ([13] and Fig. 1D) and genes involved in pyocyanin synthesis (Fig. 1D). Moreover, CzcR is also required for the positive transcriptional regulation of at least *lasI*, *rhlI* and *rhlR* quorum sensing regulator genes (Fig. 3B) and for its own *czcRS* operon and the *czcCBA* metal efflux pump operon. In order to investigate whether CzcR directly regulates the transcription of these genes, we decided to examine the recruitment of CzcR on the promoter of these targets using chromosome immunoprecipitation experiments (ChIP). For this purpose, we constructed a strain carrying an N-terminal hemagglutin-tagged (HA) version of CzcR at the *czcR* genomic locus. Western Blot analysis of this CzcR-HA tagged strain and a wild type strain confirmed that this modified version of CzcR is well-expressed in the presence of zinc and that, like in a wild type strain, OprD expression levels are repressed when CzcR-HA is induced by zinc (Fig. 5A).

As expected, ChIP experiments revealed that CzcR was able to be associated to its own promoter in the presence of zinc, the inducing metal, indicating a direct positive transcriptional *czcRS* self-regulation (Fig. 5B). Additionally, this experiment showed that CzcR was highly recruited to the *oprD* and *phzA1* promoters when the CzcR-HA strain was grown in the presence of zinc (Fig. 5B). Importantly, under these conditions, we also observed the localization of CzcR on the *lasI* promoter demonstrating a direct control of the Las system by this response regulator (Fig. 5B). CzcR was not detected on the other QS promoters, *rhlI*, *rhlR* and *lasR.* This suggested that the requirement of CzcR for QS might involve the regulation of *lasI.*

CzcR was however not detected on the promoter of *phzS* (Fig. 5B), *phzA2 and phzM* (data not shown) suggesting that this regulator also indirectly controls the expression of other genes. Altogether, these data demonstrated that in addition to its role in up-regulating its own expression and that of the CzcCBA metal efflux pump, CzcR is clearly a direct transcriptional repressor of *oprD* and *phzA1* genes. Additionally, CzcR directly positively modulates the *lasI* autoinducer synthase gene by interacting with its promoter. CzcR is therefore an important regulator controlling metal resistance, carbapenem antibiotic resistance and the Las quorum sensing system in *P. aeruginosa.*

## Discussion

*P. aeruginosa* possesses 64 sensor kinases and 72 response regulators enabling it to adapt to various and changing environments [42]. A variety of these TCS have already appeared instrumental in the regulation of both virulence processes and resistance to antimicrobial compounds [43], [44]. In a previous study, we demonstrated that the metal-inducible CzcRS TCS, involved in resistance to Zn, Cd and Co in *P. aeruginosa*, was also involved in carbapenem antibiotic resistance by repressing the OprD porin [13]. In the present work, we showed that CzcR modulates the expression of the important virulence factors pyocyanin, LasB elastase, rhamnolipids and biofilm formation. We demonstrated that, in the absence of the CzcR protein, the transcription of the two acyl-homoserine lactone synthase genes, *lasI* and *rhlI,* dropped drastically (Fig. 3B). Moreover, the positive regulatory effect of CzcR on QS is essential for the entire pathogenicity of *P. aeruginosa* in the *C. elegans* animal model (Fig. 4).

Other examples of TCS required for bacterial pathogenicity by modulating QS have been described in the literature. The most extensively studied example is the PhoQ/PhoP TCS that senses magnesium concentration. This TCS is required for the virulence of many Gram-negative bacteria such as *Shigella, Pseudomonas, E. coli, Salmonella* [3], [43], [45], [46], [47]. In *P. aeruginosa* the GacA/GacS TCS activates the transcription of two small RNAs RsmZ and RsmY that inactivates the repressor protein RsmA, thus allowing the synthesis of acyl-homoserine lactone molecules [48].

How does CzcR modulate quorum sensing? Since the LasR/3-oxo-C12-HSL complex is at the top of the QS regulatory circuit (*reviewed in* [27]), it seems possible that the effect on virulence factor production and the decrease of the Rhl and Las QS systems might be due to the lack of 3-oxo-C12-HSL autoinducer. In agreement with this, control by CzcR of the *lasI* autoinducer synthase genes seems to occur, since this regulator is clearly associated with this promoter in presence of Zn (Fig. 5B). However, the RT-PCR experiment showed that the addition of Zn does not appear to significantly enhance activation of the expression of these genes (Fig. 3). It is conceivable that the increase is below the limit of detection of our semi-quantitative RT-PCR assay. This view is consistent with the production of elastase, rhamnolipids and auto-inducers that repeatedly display a small increase in the presence of Zn (Fig. 2 and 3).

Alternatively CzcR could antagonize the effect of the numerous negative regulators such as MvaT, RpoN, RsmA or RsaL that control the expression of *lasI* [23], [28]. RsaL is a small 11 kDa protein, which binds to the *lasI* promoter and represses its expression [49], [50]. Since the *rsaL* promoter is located in the vicinity of the *lasI* promoter, a possible explanation for the control of CzcR over quorum sensing might involve RsaL. Expression of *rsaL*, however, was affected neither by Zn treatment nor by the deletion of *czcR* (data not shown), suggesting that CzcR control might involve another mechanism. The direct control of QS expression and the identification of a putative DNA binding consensus sequence will be the subject of further investigations. The effect of CzcR presented here shows that this protein could be considered as a new QS regulator involved in virulence factor expression in addition to metallic stress response and carbapenem antibiotic resistance [13].

Intriguingly CzcR regulates the expression of several genes in opposite ways: the full production of some virulence factors as well as its own expression requires CzcR, while pyocyanin production and *oprD* are repressed by CzcR (Fig. 1, 2, 3). Hence we propose that CzcR has a dual function and can act as both a repressor and an activator has it have already been observed for other response regulator [51]. CzcR is able to activate the expression of its own and of the efflux pump CzcCBA in presence of the metal signal, indicating that its activator activity on these genes might require its phosphorylation by CzcS. Unexpectedly, CzcR was able to repress the transcription of *phzA1* and to induce the transcription of several QS genes when present in low amounts in cells, in the absence of induction by heavy metals. It is thus conceivable that CzcR basal activity does not require activation of CzcR by phosphorylation. One cannot exclude the phosphorylation of CzcR *in vivo* by phosphor-donors such as acetyl phosphate as it has been observed for some response regulators [52]. However recent data suggest that even in the absence of phosphorylation, some RR might be active. In *Salmonella*, CgsD functions in its unphosphorylated form for biofilm formation, while phosphorylation of the aspartate residue reduces its activity, making it less stable *in vivo* [47]. The response regulator MtrA in *Corynebacterium glutamicum* was able to bind DNA, though the protein was presumably unphosphorylated [51].

Nevertheless we observed that expression of a wild type *czcR* allele alone in the *czcRS* deletion mutant only moderately restored the repression of the transcription levels observed in presence of zinc, where CzcR can be phosphorylated by CzcS. One reason why the mutant is unable to be fully complemented by *czcR* alone would be that CzcR needs to be phopsphorylated to be a fully active. This activity is similar to the phosphorylated MtrA protein that displays much higher DNA-binding affinity than the unphosphorylated form [51]. Furthermore, data from OmpR-type response regulators and MtrA studies indicate that phosphorylation of the conserved aspartate residue leads to dimerization in tandem which would give a more compatible structure with their binding to direct repeat DNA motifs [51], [53]. Mutation of the phosphorylated aspartate residue of the CzcR protein is under way to define the role of CzcR phosphorylation in transcriptional regulation. Altogether these data suggest that a correct balance between CzcR protein amount and CzcS ability to phosphorylate is critical for optimum CzcR activity.

With this study, we reported that a TCS regulator, CzcR, directly contributes to controlling the transcription activity of several genes even in the absence of cognate signal. This highlights a new function of TCS regulators acting as independent transcription factors. Additionally, the results presented here demonstrate that the CzcRS TCS links environmental pollutants as metals to antibiotic resistance and pathogenicity of *P. aeruginosa.* These results are of primary importance since trace metals are not only present in the environment, but also within the body and are included in the composition of numerous medical treatments. It was previously observed that zinc released from urinary catheters decreases OprD porin expression, causing carbapenem resistance [54], [55]. We have reported that the zinc released by certain catheters reaches a concentration of approximately 1 mM in urine. This amount is sufficient to induce imipenem resistance in *P. aeruginosa* [13]. Moreover, a recent study reported that bronchial secretions from patients with cystic fibrosis, who are highly susceptible to *P. aeruginosa* infections, contain higher zinc concentrations relative to the ones found in healthy individuals [56]. Deciphering the pathways by which trace metals can impact antibiotic resistance and exacerbate virulence is therefore of prime interest.

## Materials and Methods

### Bacterial Strains, plasmids and Growth Conditions

The bacterial strains and plasmids used in this study are listed in Table 1. *P. aeruginosa* strains were grown at 37°C in Luria-Bertani (LB) (US biological) [57] medium; 200 µg mL^−1^ carbenicillin was added to maintain plasmid selection as appropriate. Liquid cultures containing zinc were grown in LB medium supplemented with 0,5 mM of zinc. Liquid cultures were grown on a rotary shaker in 200-ml Erlenmeyer flasks containing 25 ml of LB medium. To enhance pyocyanin production on solid plates, *Pseudomonas* Agar King A medium (Biolife) was used. The complementation of the Δ*czcRS* strain was performed using the pSWT or pRWT [13] containing *czcS* and *czcR* gene cloned under the control of the IPTG-inducible *tac* promoter in plasmid pMMB66EH [58]. This leaky promoter allowed, without adding IPTG, a weak expression similar to the wild type in the absence of the inducing metal (data not shown) and, using IPTG, a level similar to that observed in the wild type under zinc induced condition, excluding a dosage effect (Fig. 1D).

**Table 1:** Bacterial strains and plasmids used in this study.

Strain or plasmid	Relevant characteristic(s)	Reference or source
***P.aeruginosa***		
Wild type	PAO1 wild type	wt, laboratory collection
Δ*czcRS*	PAO1 Δ*czcRS*	[12]
CzcR-HA	PAO1 *czcR* gene fused with 3HA on N-terminus	This study
PAO-JP2(pECP61.5)	*ΔlasI*::Tn501/Δ*rhlI* mutant PAO-JP2 carrying plasmid pECP61.5 [63] which contains an *rhlA – lacZ* fusion together with the *rhlR* gene expressed from the strong *tac* promoter (P_tac_).	[63]
***E.coli***		
MG4λl_1_4 (pPCS1)	MG4 strain:Δ(argF-lac)U169 zah-735::Tn10 recA56 srl::Tn10, containing the bacteriophage λI14: *lasI-lacZ* and the pPCS1 plasmid: Amp^r^, lasR	[33]
**Plasmids**		
pMMB66EH	broad host range expressing vector, Cb^r^	[58]
pEX18Ap	gene replacement vector, Amp^r^	[61]
pRWT	*czcR* gene on pMMB66EH, Cb^r^	[13]
pSWT	*czcS* gene on pMMB66EH, Cb^r^	[13]
pHA1	triple HA epitope on pKS, Amp^r^	[59]

### Construction of a CzcR-HA tagged strain

CzcR was tagged with a triple hemagglutinin tag (HA) on its N-terminus part. To create this construct, the triple HA epitope was first isolated from the pHA1 plasmid by digestion with SmaI and NruI restriction enzymes [59]. A 600 bp fragment containing the *czcR* promoter and the beginning of *czcR* coding region was generated by PCR using proczcC-F primer (5′-ccaggcagagtcccatcagtagc) and primer #127 which contains a NruI (underlined) restriction site (5′-ttcgataataaggatgcg**tcgcga**catgttcgcccctatata). Another 900 bp fragment, corresponding to the beginning of *czcR* and *czcS* genes was generated using primer #126, which includes a NruI restriction site (5′-tatataggggcgaacatg**tcgcga**cgcatccttattatcgaa) and primer #124, which has a BamHI restriction site inside (5′-cg**ggatcc**tgcagggcatgcgcc). A second round of PCR was performed using proczcC-F and #124 as primers and the two overlapping previously generated fragments as template. The 1500 bp product containing a NruI site just after the first methionine and a BamHI site at the 3′-end was then digested with BamHI and cloned into pKS-Bluescript digested by ScaI/BamHI. The triple HA epitope was inserted within the NruI site. After a test PCR to verify correct tag orientation, a 1620 bp fragment containing the whole construct was isolated using SmaI/BamHI restriction enzymes. This fragment was then cloned into pEX18Ap and transformed by electroporation in a PAO1 wild type strain [60]. Integration of the generated *czcR-HA* cassette into the chromosome was performed by selection on 200 µg mL^−1^ carbenicillin. Clones were restreaked onto LB containing 5% sucrose to select for double recombination events [61]. Finally, correct integration was verified by PCR.

### Pyocyanin quantitation assay

Pyocyanin quantification was performed using the assay based on absorbance at 520 nm in an acidic solution [62]. Briefly, 5 ml supernatant from a stationary phase culture, in King A medium to maximize pyocyanin production, was mixed with 3 ml of chloroform. Pyocyanin from the organic phase was then extracted with 1 ml of 0.2 N HCl, giving it a pink to deep red color due to pyocyanin. Absorbance was measured at 520 nm. Concentrations, expressed as micrograms of pyocyanin produced per milliliter of culture supernatant, were determined by multiplying the optical density at 520 nm (OD_520_) by 17.072 [62]. The experiment was conducted three times in three independent experiments.

### Determination of autoinducer concentrations

Filtered culture supernatants in early stationary phase (OD_600_ of 1) were extracted with ethyl acetate. Autoinducer (AI) concentrations were determined in bioassays as previously described, by using *P. aeruginosa* PAO-JP2(pECP61.5) for C4-HSL [63] and *E. coli* MG4λI_1_4(pPCS1) for 3-oxo-C12-HSL [33] with the following changes. *E. coli* cultures harboring *lacZ* fusion plasmids were grown overnight at 37°C with vigorous shaking in LB medium containing 100 µg ml^−1^ ampicillin, then inoculated into LB medium with ampicillin to a starting optical density at 600 nm (OD_600_) of 0.08. 1 ml of the culture was distributed to each AI-containing tube and incubated with shaking to an OD_600_ of 0.7. β-galactosidase activity was then determined as previously described [64]. *P. aeruginosa* cultures were grown overnight in liquid PTSB medium with 200 µg ml^−1^ carbenicillin and diluted into PTSB without antibiotics to an OD_600_ of 0.3 before addition of 1 ml of this culture to the AI-containing tubes. β-galactosidase activity was then analyzed when the culture reached an OD_600_ of approximately 0.9. Each assay was performed three times in three independent experiments.

### Elastase and rhamnolipid production assays

The LasB elastase activity of bacterial suspensions was determined with the Elastin-Congo red (ECR; Sigma, St. Louis, MO) assay [32], [65] with minor modifications. Briefly, 50 µl aliquots of filtered bacterial supernatant of a 21 h culture were added to 900 µl of ECR buffer (100 mM Tris, 1 mM CaCl_2_, pH 7.5) containing 20 mg of ECR and then incubated with shaking at 37°C for 18 h. Insoluble ECR was removed by centrifugation and the absorption of the supernatant was measured at 495 nm and normalized according to cell density (OD_600_). LB medium was used as a negative control. Elastase activity was assayed three times in three independent experiments.

Rhamnolipid production was measured on plates by inoculating strains in M9-based [57] agar plates supplemented with 0.2% glycerol (vol/vol), 2 mM MgSO_4_, trace elements, 5 mM KNO_3_ instead of NH_4_Cl as nitrogen source, 0.0005% (vol/vol) methylene blue, and 0.02% (vol/vol) cetyltrimethylammonium bromide [31]. Plates were incubated at 37°C until a blue halo appeared around the colony.

### Biofilm formation assay

A static biofilm assay with 15 ml polypropylene tubes was used, as previously described [66]. Overnight precultures in LB medium were diluted to an OD_600_ of 0.05 in 3 ml of M9 minimal medium containing 0.04% glucose as carbon source [57] into polypropylene tubes. Tubes were incubated for 8 h at 37°C without shaking. The culture, containing planktonic cells, was removed. *P. aeruginosa* cells adherent to the tube were considered as a biofilm. Tubes were washed once with distilled water and biofilms were stained with crystal violet (CV, 1% in water) for 30 minutes. CV was discarded and tubes were rinsed once with water to remove excess of dye. The stained biofilms were resuspended in 33% acetic acid and their density was evaluated by measuring the OD_590_ of the suspensions normalized for bacterial density (OD_600_). The measurement was performed three times in three independent experiments.

### *C. elegans* killing assay

The *C. elegans* killing assay was performed according to [40] using the *C. elegans* wild-type strain N2 Bristol [67]. Briefly, *C. elegans* were maintained at 20°C under standard conditions [67]. *P. aeruginosa* strains to be tested were cultured overnight and spread on PGS plates (1% bacto-peptone, 1% Nacl, 1% glucose, 0.15 mM sorbitol, 1.7% Bacto agar) complemented with 75 µM 5-fluoro-2′-deoxy-uridine (FUdR) to prevent eggs from hatching. The plates were incubated 24 hours at 37°C. 50 worms were then transferred to the lawn of the bacterial strain, incubated at 25°C and examined under a microscope at 24 h intervals for viability. Worms that did not move when touched with sterile inoculators were considered as dead. The *E. coli* OP 50 was used for control as a non-pathogenic strain. The percentage of living worms, scored in three independent experiments, was plotted using GraphPad Prism 5 software. The statistical relevance of the results was determined using the Log-rank (Mantel-Cox) and Gehan-Breslow-Wilcoxon tests.

### Western Blot Analysis

Overnight precultures of a wild type *P. aeruginosa* PAO1 and CzcR-HA strain grown in LB were diluted to an OD_600_ of 0.05 in LB medium with or without 0.5 mM ZnCl_2_. Cultures were grown at 37°C on a rotary shaker to an OD_600_ of 2.5. 1 mL of culture was spun down in a microfuge and total proteins were solubilized to a concentration of 2 mg mL^−1^ by sonication in the appropriate volume of 1× β-mercaptoethanol gel-loading buffer (an OD_600_ of 1 gives 0.175 mg/ml of protein). Samples were boiled for 5 min, then centrifuged for 10 min in a microcentrifuge to remove bacterial debris before loading. 15 µl (30 µg) of total protein were separated on a 12% SDS-acrylamide gel and transferred to nitrocellulose membrane. Blots were incubated with anti-OprD, anti-HA and anti-Hsp70 antibodies and revealed by chemiluminescence. All antibody incubations and washes were performed in TBS-T (20 mM Tris, 137 mM NaCl, 0.1% Tween 20, pH 7.6) supplemented with 5% powdered milk.

### Chromosome immunoprecipitations (ChIP)

ChIP experiments were performed as previously described [68]. Immunoprecipitated DNA was quantified by real-time PCR using a Sybr Green mix (IQ ™SYBR Green Supermix Bio-Rad) according to the supplier's specifications. The DNA samples were diluted 10-fold, and 5 µl of this dilution served as the template in the PCRs that were performed in duplicate for each gene and sample. The primers used are listed in Table 2. Each amplification was normalized by the *oprF* value of the corresponding immunoprecipitation. The results, expressed as fold increase compared to *oprF,* represented the average of three independent experiments and the standard deviations are indicated.

**Table 2:** Primers used for qRT-PCR.

Amplicon	Primer	Sequence (5′to 3′)	Position	length
***Coding region:***				
*oprD*	oprD1	ATCTACCGCACAAACGATGAAGG	772	156
	oprD2	GCCGAAGCCGATATAATCAAACG	927	
*czcR*	czcR1	GTCATCACCCGGACGCAGATCAT	502	153
	czcR2	GTAGCCGACGCCGCGAATGGTAT	654	
*czcS*	czcS1	TACGCCAGCTCTCGCAGTTCTCC	740	201
	czcS2	TGTCCACCTGCACCAGGAACAGC	940	
*oprF*	oprF1	GGTTACTTCCTGACCGACGA	172	664
	oprF2	TCGGTGTTGATGTTGGTGAT	836	
*phzA1*	249	AACCACTTCTGGGTCGAGTG	283	202
	250	GTGGGAATACCGTCACGTTT	485	
*phzA2*	343	CGAGAGTACCAACGGTTGAA	4	481
	250	GTGGGAATACCGTCACGTTT	485	
*phzM*	259	CAAGTTGTTACCGGGGAATG	40	172
	260	AGATCTCGAAGGCCACCAG	211	
*phzS*	269	GGAAAGCAGCAGCGAGATAC	102	206
	270	AGTACTGCGGATAGGCGTTG	307	
*lasI*	311	CTACAGCCTGCAGAACGACA	390	168
	312	ATCTGGGTCTTGGCATTGAG	557	
*lasR*	334	ACGCTCAAGTGGAAAATTGG	33	247
	335	GTAGATGGACGGTTCCCAGA	279	
*lasB*	301	AAGCCATCACCGAAGTCAAG	260	230
	302	GTAGACCAGTTGGGCGATGT	489	
*rhlI*	309	CTCTCTGAATCGCTGGAAGG	13	240
	310	GACGTCCTTGAGCAGGTAGG	252	
*rhlR*	307	AGGAATGACGGAGGCTTTTT	4	231
	308	CCCGTAGTTCTGCATCTGGT	234	
*rhlA*	299	CGAGGTCAATCACCTGGTCT	279	230
	300	GACGGTCTCGTTGAGCAGA	489	
***Promoter:***				
*pczcR*	348	GCAACCTTCGAAGAGACTGG	−295	236
	349	AAGTTACATTTCGGGCGTTG	−60	
*poprD*	poprD R	CGCAGATGCGACATGCGTCA	−178	101
	poprD L	GGCGCTCCACTTCATCACTT	−77	
*pphzA1*	257	TACCCTGTCTGGCACCTACC	−402	185
	258	ACCTGTCGGTAATGGATTCG	−218	
*pphzS*	338	GTTCGAACTGTGCCTGGAGT	−354	250
	339	GTCATTCGCCCTACGAACC	−105	
*poprF*	poprF R	TTGCGAACGCTGTCGGTGAA	131	215
	poprF L	GCGGGAAGTTCTGATAAACT	−84	
*plasI*	322	ATAGGGAAGGGCAGGTTCTC	−291	169
	323	TCAGAGCAATGGCTTCACAC	−123	
*prhlI*	320	TTCCACCACAAGAACATCCA	−266	174
	321	CACACATGAGGGGGAAGACT	−93	
*plasR*	lasR P1	ACTAGGTGCATCAAACGC	−232	200
	lasR P2	GCCAAATATGGATTCGGC	−33	
*prhlR*	316	GAATTGTCACAACCGCACAG	−225	203
	317	ATGCATCACAGCAGAATTGG	−23	

### Semi quantitative RT-PCR analysis

For RNA isolation, strains were cultured in King A medium for the experiments of Fig. 1D or LB media for the experiments of Fig. 2D. Overnight precultures were diluted to an OD_600_ of 0.05 and grown for 6 hours at 37°C (OD_600_ of 2.5). 0.5 ml of this culture, was added to 1 ml of RNA Protect bacteria solution (Qiagen, Hildesheim, Germany), and total RNA was isolated with RNeasy columns (Qiagen, Hildesheim, Germany) according to the supplier's instructions. Residual DNA was eliminated by DNase treatment using 20 units of RQ1 RNase-free DNase (Promega). After removal of DNase by phenol/chloroform extraction, RNA was precipitated, and the pellet resuspended in 30 µl of RNase-free H_2_O. For cDNA synthesis, 500 ng of RNA was reverse-transcribed using random hexamer primers (Promega) and Improm-II reverse transcriptase (Promega) according to the supplier's instructions. PCR amplifications were performed using standard procedures with 30 cycles, except for *czcS* and *phzA2* for which the amplification was carried out with 27 cycles and *oprD*, *oprF, czcR, phzA1, phzS*, for which the amplification was carried out with 26 cycles to avoid over-amplification. The primers used for PCR amplification are listed in Table 2. Each analysis was performed at least three times from three independent cultures. A representative analysis is presented.
